# Development of Biomarkers and Molecular Therapy Based on Inflammatory Genes in Diabetic Nephropathy

**DOI:** 10.3390/ijms22189985

**Published:** 2021-09-15

**Authors:** Amit K. Maiti

**Affiliations:** Department of Genetics and Genomics, Mydnavar, 2645 Somerset Boulevard, Troy, MI 48084, USA; akmit123@yahoo.com or amit.maiti@mydnavar.com; Tel.: +1-248-379-3129

**Keywords:** diabetes, nephropathy, inflammation, genes, biomarker, precision therapy

## Abstract

Diabetic Nephropathy (DN) is a debilitating consequence of both Type 1 and Type 2 diabetes affecting the kidney and renal tubules leading to End Stage Renal Disease (ESRD). As diabetes is a world epidemic and almost half of diabetic patients develop DN in their lifetime, a large group of people is affected. Due to the complex nature of the disease, current diagnosis and treatment are not adequate to halt disease progression or provide an effective cure. DN is now considered a manifestation of inflammation where inflammatory molecules regulate most of the renal physiology. Recent advances in genetics and genomic technology have identified numerous susceptibility genes that are associated with DN, many of which have inflammatory functions. Based on their role in DN, we will discuss the current aspects of developing biomarkers and molecular therapy for advancing precision medicine.

## 1. Introduction

Diabetes Nephropathy (DN) is a chronic kidney disease in which increasing abnormalities are developed when filtering waste and extra water from the body due to long-term hyperglycemia and hypertension. Deterioration of kidney function occurs both in Type 1 (juvenile) and Type 2 diabetes when high blood sugar levels damage micro blood vessels in the kidney. Kidney disease occurs in one of four diabetic patients [1]. In 2015, the International Diabetic Federation estimated that the prevalence of diabetes was 8.8% from ages 20 to 79 years, affecting a population of approximately 440 million people [2]. This is predicted to grow to over 550 million people by the year 2035 [3]. As diabetes is a world epidemic, a vast number of people are considered to be affected by DN.

Until recently, the extent of DN was measured by the presence of albumin in the urine of diabetic patients (>−300 mg/mL) and defined as albuminuria or proteinuria. However, recent studies show that sufficient glomerular damage happens before the increase in albumin in the urine. Thus, new biomarkers are needed to assess the early stages of DN, as well as to measure the extent of kidney damage [4]. A proteomic study confirmed Nonalbumin Proteinuria (NAP) with the presence of alpha 1 microglobulin, globulin, nephrin, etc., as the sensitive indicators of early tubular damages [5]. When precipitation of the morning urine was analyzed with subsequent resolution by 2D gel electrophoresis, it identified a protein, kininogen-1, involved in the kalicranin–kinin system as a potential indicator, but it has not been validated in a large cohort [6].

Diabetic Kidney Disease (DKD) is the leading cause of End-Stage Renal Disease (ESRD) and is associated with increased mortality due to cardiovascular abnormalities secondary to diabetes. Although the difference between DKD and DN is difficult to distinguish clinically, DKD is used for a wide spectrum of changes in pathologies in the kidney, whereas DN is defined with distinct histological gradation. In 47 renal tissues of DKD patients, 46 types of inflammatory cells, adhesion molecules, and distinct cytokines are detected in nephropathy. The current understanding of DKD recognizes the involvement of metabolic abnormalities, hemodynamic changes, Renin–Angiotensin System (RAS) activation, and oxidative stress. While many of these approaches have been clinically implemented to slow the acceleration of DKD, current management is insufficient, both for preventing or for halting disease progression. There is a growing appreciation for the role of inflammation in modulating the process of DKD [7]. In nephropathy, the infiltration of inflammatory cells with the expression of adhesion molecules and cytokines are detected in renal tissue of DKD patients. An influx of macrophages is a principal feature during the progression of kidney diseases [8] and is highly correlated with the decline in the Glomerular Filtration Rate (GFR).

Thus, the initial characterization of kidney and renal tubule damage is of prime importance in DN, as is the development of continuous improvements in therapeutic aspects. Recent advances in genetic and genomic technology have opened new avenues in developing biomarkers for the early detection of kidney damage and new therapies. In this review, we discuss the advances in developing biomarkers and the therapeutic potential of genes or proteins that are genetically identified to play a role in inflammation. We will provide an overview of the advantages over traditional and current drug developments to detect early abnormalities and their possible role in therapeutic interventions.

## 2. Brief Overview of Stages of Diabetic Nephropathy

The kidney consists mainly of glomeruli, tubules, and vessels. Each component is deteriorated during the progression of kidney diseases.

(a). *Glomerular lesions*

Tervaert et al., 2010 [9] classified four classes of hierarchical glomerular lesions with a separate evaluation for degrees of interstitial and vascular involvement.

Type 1: Glomerular Basement Membrane (GBM) Thickening.

GBM thickening is defined by the deviation from normal GBM thickness. It is an early change in Type 1 and Type 2 DN that increases with duration of disease.

Types 2: Mild (IIa) or Severe (IIb) Mesangial Expansion.

Mild or severe mesangial expansion is based on whether the expanded mesangial area is smaller or larger than the mean area of a capillary lumen.

Types 3: Nodular Sclerosis (Kimmelstiel–Wilson lesions).

Kimmelstiel–Wilson lesions appear in Type 1 and Type 2 diabetes as focal, lobular, and round to oval mesangial lesions with an acellular hyaline/matrix core. They are rounded peripherally with crescent-shaped mesangial nuclei.

Types 4: Advanced Diabetic Glomerulosclerosis.

Glomerulosclerosis in DN is the end point of multifactorial mechanisms that lead to excessive accumulation of Extracellular Matrix (ECM) proteins. These proteins mainly consist of collagen types I, III, and IV and fibronectin in the mesangial space. They contribute to mesangial expansion and the development of Kimmelstiel–Wilson lesions resulting in glomerulosclerosis.

(b). *Interstitial abnormalities*

Two types of interstitial abnormalities are characterized.

Types 1: Interstitial Fibrosis and Tubular Atrophy (IFTA)

Interstitial Fibrosis and Tubular Atrophy (IFTA) follow glomerular changes in DN. IFTA is calculated as the percentage of changes of interstitium and tubules from the total involved area. It ultimately leads to ESRD.

Types 2: Interstitial inflammation

Inflammatory interstitial infiltrates comprise T lymphocytes and macrophages. The inflammatory infiltrate also occurs in atrophic tubules and in other areas.

(c). *Vascular lesions*

Vascular lesions are divided into three types.

Types 1: Arteriolar hyalinosis

Arteriolar hyalinosis is defined as the thickening of the arteriolar walls by the deposition of hyaline material. It depends on clinical parameters without distinguishing between efferent and afferent arterioles during disease progression.

Types 2: Presence of large vessels

Osterby et al., 2019 [10] used a so-called “matrix-to-media ratio” to define the enlargement of vessels. This ratio is increased in patients with microalbuminuria, suggesting that arteriolar matrix accumulation occurs early in the course of DN. These abnormalities might lead to less albumin secretion in the urine.

Types 3: Arteriosclerosis

Arteriosclerosis is the most severely affected artery and can be defined as the scores of thickenings. Assignment of scores is used as 0 with no intimal thickening, 1 with intimal thickening, and 2 with medial thickening. Isolated or significant medial thickness may be associated with concurrent hypertension.

(d). *Other glomerular lesions*

“Insudative lesions” are defined by the intramural accumulations of imbibed plasma proteins and lipids within renal arterioles, glomerular capillaries, Bowman’s capsule, or proximal convoluted tubules [11]. These lesions are mainly found in more advanced stages of nephropathy associated with proteinuria.

## 3. Specialized Cells in Kidney and Renal Systems

### 3.1. Specialized Cells

The kidney consists of microanatomically distinct cells with specific functions. These are kidney glomerulus parietal cells, kidney glomerulus podocytes, kidney proximal tubule brush border cells, Loop of Henle thin segment cells, thick ascending limb cells, kidney distal tubule cells, collecting duct principal cells, collecting duct intercalated cells, and interstitial kidney cells. Although each cell type has an important role in maintaining the renal system, glomerular podocyte structure and function are reported to be associated with many genes. Thus, considerable efforts are directed toward the development of biomarkers and therapy of DN by manipulating genes or proteins that modulate podocyte structure and function.

### 3.2. Structure and Function of Glomerulus Podocyte

**Figure 1:**
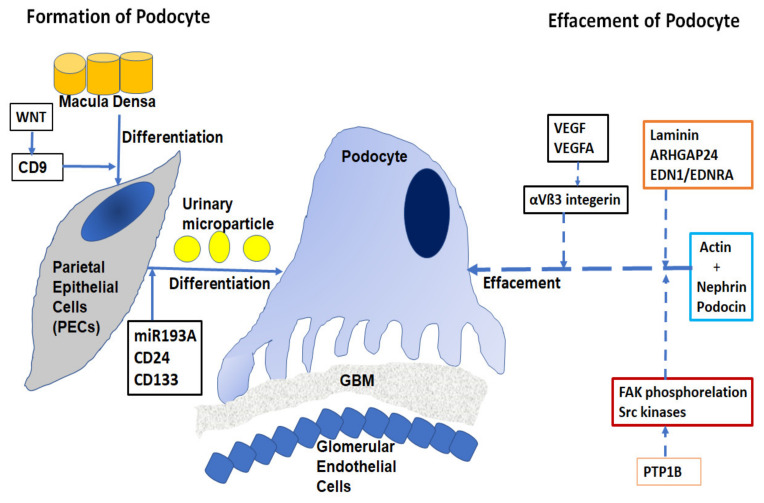
Podocyte–glomerular junction. GBM—Glomerular Basement Membrane. The macula densa differentiates into Parietal Epithelial Cells (PECs) using CD9, which is activated by WNT signaling genes/proteins. PECs differentiate into podocytes. miR193a plays a critical role in this process, whereas podocyte effacement is a complex procedure involving a series of proteins/genes, mainly with actin reorganization activated by a group of genes/proteins.

The pathogenesis of proteinuria in nephrotic diseases involves the function of the podocyte epithelial cells [12]. The filtration barrier has three layers: the glomerular epithelium, the basement membrane, and the slit diaphragm formed by the foot processes of the podocytes. The slit diaphragm is the final barrier that prevents the passage of proteins and large molecules into the urinary filtrate. Podocytes are typical epithelial cells (Figure 1) that comprise three separate structural and functional elements: a large cell body, major extending processes, and foot processes [13].

When podocytes are injured, they undergo a process of effacement and lose their structure. Effacement is associated with proteinuria, especially in Focal Segmental Glomerulosclerosis (FSGS) and diabetes. Podocyte depletion can also be correlated with the development of glomerular sclerosis and Chronic Kidney Disease (CKD) [14].

Approximately 40 genes have been implicated in the development of FSGS and related nephrotic diseases [15,16], including mutations in nephrin, podocin, TRPC6, actin, INF2, WT2 and, laminin/integrin receptors. Effacement of podocytes is caused by the breakdown of the actin cytoskeleton of the foot processes, allowing podocytes to be dynamic in nature with a rapidly changing ability required in filtration processes [17]. The cortical actin network binds with specialist proteins, such as nephrin and podocin of the slit diaphragm at the unique tethering points. Abnormalities of nephrin caused by mutations in the NPHS1 gene have been implicated in the autosomal recessive congenital nephrotic syndrome in a Finnish population [18]. Deletion of nonreceptor phosphatase PTP1B in podocytes conferred protection from injury and consequently, overexpression of PTP1B resulted in increased FAK phosphorylation and activity of Src kinases [19]. Laminin regulates focal adhesions, the glomerular slit diaphragm, actin-binding proteins, and actin-regulatory proteins such as small GTPases of the Rho/Rac/Cdc42 family [20]. Small GTPases are long known as important regulators of actin dynamics in podocytes. The GTPase-Activating Protein (GAP) Rho-GAP 24 (Arhgap24) was observed to be upregulated during podocyte differentiation. The crosstalk signaling between podocyte dysfunction and depletion in glomerulosclerosis is mediated by Endothelin-1 (EDN1)/Endothelin Receptor Type A (EDNRA)-dependent mitochondrial dysfunction [21]. Dysregulation of VEGF expression within the glomerulus has been reported in a wide range of nephrotic diseases [22]. The role of podocyte VEGFA in regulating αVβ3 integrin signaling in mouse GBM has been shown in vivo. αVβ3 integrin plays an important role in angiogenesis and in hypertension-induced vascular remodeling in the kidney. However, the mechanism of nephrin focal adhesion complex proteins, especially their post-translation modifications under different conditions of VEGF signaling, is unclear [23].

## 4. Role of Autophagy in Developing DN

### 4.1. Mechanism

Autophagy regulates many critical aspects of normal and disease conditions in the kidney. Autophagy is triggered in Streptozotocin (STZ)-induced early diabetic rats with associated renal hypertrophy by insulin replacement therapy or islet transplantation [24]. This subsequently facilitates the discovery of therapeutic targets for the prevention and treatment of kidney diseases [25]. Under diabetic conditions, three major nutrient-sensing signal pathways negatively modulate autophagic activities through activation of mTORC1 and inhibition of the AMPK and SIRT1 pathways. However, autophagy mediated by these pathways may not be the sole cause of DN, and the contribution of these pathways to develop DN is not fully understood.

#### 4.1.1. mTORC1 Pathway

Podocyte-specific activation of mTORC1 results in many features of DN, such as mesangial expansion, GBM thickening, podocyte loss, and proteinuria in nondiabetic mice. mTORC1 inhibition ameliorates diabetic changes such as renal hypertrophy in db/db mice [26]. Chaperone-Mediated Autophagy (CMA) is a lysosomal proteolytic pathway that needs LAMP-2A in the mTORC pathway. CMA target protein pax2 is critical for nephron branching during renal development [27]. Cathepsin A knockout mice have a similar phenotype as the severe human infantile form of death from renal failure at an early age [28]. These knockout mice lacking LAMP-2A have constitutively active CMA. Increased CMA in the kidney cortex, as well as decreased LAMP-2A, was also observed in STZ-induced early diabetic rats with renal hypertrophy [27]. These findings suggest that activation of the mTORC1 pathway has an important pathogenic role in DN.

#### 4.1.2. AMPK Pathway

Adenosine Monophosphate (AMP)-Activated Protein Kinase (AMPK) is a nutrient-sensing kinase. It is activated upon phosphorylation by several upstream kinases, including Liver Kinase B1 (LKB1), Calcium/Calmodulin-Dependent Kinase β (CaMKKβ), and TGF-β-Activated Kinase 1 (TAK1) [29]. Both CaMKKβ- and TAK1-mediated activation of AMPK has been implicated in AMPK-mediated autophagy induction in kidney cells.

#### 4.1.3. SIRT1 Pathway

Silent Information Regulator T1 (SIRT1), a NAD+-dependent deacetylase, is among the major nutrient-sensing pathways implicated as a positive regulator of autophagy. SIRT1 can interact with FoxO3, resulting in enhanced expression of BCL2/Bnip3 and promoting autophagy in renal cells [30]. An age-dependent decline in Sirt1 activity accelerates premature aging with renal phenotypes [30] mainly due to inhibition of autophagy, leading to DN.

### 4.2. Autophagy in Podocytes

Podocytes have a high level of basal autophagy that may serve as a mechanism for their maintenance of cellular homeostasis [31]. When mice with podocyte-specific deletion of the Atg5 gene are treated with puromycin aminonucleoside or adriamycin, more severe albuminuria, loss of podocytes, and glomerulosclerosis are observed compared to control mice [31].

### 4.3. Autophagy in Mesangial Cells

Autophagy contributes to the survival of mesangial cells. Under serum deprivation, when autophagy is induced by TGF-β1 in mesangial cells via TAK1 and PI3K-Akt, cell survival is increased by inhibiting apoptosis [32].

### 4.4. Autophagy in Glomerular Epithelial Cells

Xavier et al., 2010 demonstrated that in glomerular epithelial cells, BAMBI, a competitive receptor antagonist for the TGF-β receptor family, is regulated by autophagy [33].

### 4.5. Mitophagy

In mitophagy, an accumulation of fragmented mitochondria is found in the renal cortex in both humans and animals with DN. Hyperglycemia is thought to promote oxidative stress during diabetic complications in DN [34]. Increased ROS, a mitochondrial product, has been widely observed in diabetic kidney cells, leading to ATP depletion in the renal cortex [35].

## 5. Developing Biomarkers in DN

Classical biomarkers such as high albumin, creatinine, GFR, and Serum Uric Acid (SUA) [36] that are currently being used in clinics do not optimally predict the progression of DN. In the microalbuminuria stages, multifactorial therapy, such as blood pressure, lipid, glycemic control with smoking cessation improved renal function [37]. In recent years, the quests for developing both prognostic and surrogate endpoint biomarkers for advanced DN and ESRD have received major investments. However, no novel biomarkers are in routine use now in clinics or in clinical trials. A wide range of biomarkers is simultaneously being developed considered to be the most useful biomarkers for DN [38].

### 5.1. Biomarkers Using Biochemical Pathway

Recent studies suggest that microalbuminuria is a poor diagnostic marker of DKD [39]. Most patients with Type 2 diabetes do not manifest excessive urinary albumin loss but still develop CKD and ESRD. Indeed, in the United Kingdom Prospective Diabetes Study (UKPDS) cohort, 28% of patients developed moderate to severe renal impairment; 14% did not have precise albuminuria [40], and 11% of patients with Type 1 diabetes in the Diabetes Control and Complications Trial (DCCT) developed proteinuria with GFR decline [41]. Albuminuria and GFR decline might be independent manifestations of DKD because some patients show albuminuria, while others only show GFR decline without albuminuria [40]. T1D patients with normoalbuminuria and low GFR show classic changes of DKD in histological sections. In contrast, some T2D patients have heterogeneous changes with normal or near-normal histology with disproportionately severe interstitial or tubular or vascular damage, while others have no or only mild diabetic glomerular changes [42]. Serum cystatin C based eGFR has been proposed to be advantageous because, unlike creatinine, it is unrelated to muscle mass. Several biomarkers are being developed, such as urinary angiotensinogen and ACE2 [43], plasma copeptin [44], plasma endostatin, serum amyloid [45], urinary NGAL, cystatin C, serum TNFR1, and TNFR2 [46]. Agarwal et al., 2014 [47] observed that urinary C-terminal FGF-2 showed the strongest association with ESRD, whereas plasma VEGF showed association with the composite outcome of death and ESRD.

### 5.2. Using the Mass Spectrometry Method

Argile et al., 2013 [48] used a mass-spectrometry-based method to combine 273 urinary peptides to create a score that has high accuracy in determining the cross-sectional classification of eGFR. Based on that, they developed a commercial test with Mosaique Diagnostics (http://mosaiques-diagnostics.de/mosaiques-diagnostics, accessed on 29 July 2021).

### 5.3. Other Proteomics Method

A nested case–control plasma proteomics study yielded kininogen and kininogen fragments as predictors of renal function decline [49].

### 5.4. miRNA Biomarker in DN

Involvement of miRNAs in kidney diseases is assessed in severe renal phenotypes in mice with podocyte-specific deletion of the Dicer gene. Phenotypes of these mice include proteinuria, podocyte foot process effacement and apoptosis, glomerulosclerosis, and tubulointerstitial fibrosis with renal failure [50]. The in vivo involvement of miR-192 in the pathogenesis of DN was demonstrated by both miR-192 gene knockout mice and mice treated with miR-192 inhibitor [50,51]. Increased miR-21 expression is documented in renal transplant patients with fibrotic kidney disease and in the urine of fibrotic patients with IgA nephropathy [52]. The miR-200 family of miR-200a, miR-200b, miR-200c, miR-429, and miR-141 plays a crucial role in maintaining epithelial differentiation, suggesting their role in antifibrotic functions in DN [53]. Assmann et al., 2018 [54] identified six miRNAs that were consistently dysregulated in DKD patients compared to controls: miR-21-5p, miR-29a-3p, miR-126-3p, miR-192-5p, miR-214-3p, and miR-342-3p. Expression of miR-192 and its downstream miRNAs, miR-216a, miR-217, and miR-200 family were all effectively inhibited by Lipid Nanoparticle Attachment (LNA)-modified anti-miR-192 in the renal cortex of STZ-injected diabetic mice [55]. Therefore, inhibiting disease-inducing miRNAs in patients during the early stages of DN could be clinically feasible to prevent the progression of renal disorders.

### 5.5. LncRNA Biomarker in DN

LncRNA LINC01619 has been shown to regulate miR-27a/FoxO1 and ER stress-mediated podocyte injury in DKD [56]. LncRNAs (HypERlnc) are regulated by hypoxia-induced ER stress that is identified in pericytes [57]. LncRNA 1700020I14Rik alleviates cell proliferation and fibrosis in DKD through miR-34a-5p/Sirt1/HIF-1α signaling pathway [58]. LncRNA Tug1 plays an important role in mitochondrial bioenergetics in DN through PGC-1α [59]. Thus, LncRNAs can be developed as biomarkers for DN, although more reports are needed to show the correlation of LncRNA expression with kidney diseases and their various stages of ailments, which will provide a more precise diagnosis for patients [60].

### 5.6. Developing Biomarkers Based on Autophagy

The most effective treatment strategy of DN is an intensive treatment to strictly control glycemia and blood pressure using Renin–Angiotensin System (RAS) inhibitors. Whether mTORC1 inhibition is safe and effective for all patients with DN is still debatable. Excessive mTORC1 inhibition also led to podocyte dysfunction [61]. When inactivated dephosphorylated AMPK is reversed by metformin or resveratrol, the attenuation of diabetic glomerular and tubular injury is observed [62]. 5-aminoimidazole-4-carboxamide ribonucleotide and metformin can improve renal lesions in DN mice models. It might improve DN through the restoration of autophagy activity in diabetic kidneys. Taken together, these findings suggest that nutrient-sensing signal proteins are strong targets for modifying autophagy activity in the kidney.

## 6. Epidemiology and Genetic Factors in DN

Familial aggregation, candidate gene approach, and Genome-Wide Association Studies (GWAS) have been widely used to identify genes that are associated with DN. Familial aggregation in DN is well observed, and familial clustering results from shared genes, environmental exposure, or their combination [63]. Several genes that predispose to diabetes have recently been identified with genetic susceptibility to the microvascular complication of nephropathy in individuals with both T1D and T2D. Polymorphisms in numerous genes are identified to play important roles in kidney diseases (reviewed by Tziastoudi et al., 2020 [64]; Wei et al., 2018 [65]). Some of these genes are ACE1, MTHFR, TGFB1, UMOD, PRKAG2, Apolipoprotein E, Angiotensinogen, PRKAA2, MyD88, IRAK4, TRAF6, HS6ST1, RAB38 ACACB, PFKFB2, TNFα, IL6, IL10, IFN Gamma (IFNG), CCR5, IL8, CCL2, MCP-1, RREB1, NPHS1, NOS3, CD2AP, TRPC1, SOD1, IL1RN, GFPT2, PRKCB1, ELMO1, VEGFA, VDR, MIF1, IL1B, TNFRSF19, MMP3, MMP12, IL12RB1, EPO, GHRL, PRKCE, IL100, IL1A, ADIPOQ, IGF1, BCL2, and GCKR.

## 7. Developing Therapy Based on Mutations in Inflammatory Genes in DN

Among the genes that are identified to be associated with DN are several inflammatory genes [66]. These inflammatory genes are TGFB1, BCL2, IGF1, ADIPOQ, IL1A, IL1RN, IL8, IL1B, MIF1, VDR, IL10, IL6, IL18, MCP-1/CCL2, VEGFA, ELMO1, IFNG, TNFα, MMP3, MMP9, MMP12, EPO, GHRL, TRAF6, and IRAK4.

TGF-ß and VEGFA: Transforming Growth Factor-Beta (TGF-β) is a cytokine that is widely associated with the development of fibrosis in DN. TGF-β and its downstream SMAD signaling play a critical role in the development and progression of renal fibrosis. TGF-β acts as a principal effector mediating podocyte injury and apoptosis [67]. In the acute phase of glomerular injury, it induces ECM, cell differentiation and proliferation, apoptosis, and Epithelial-to-Mesenchymal Transition (EMT) [68]. In podocytes, CTGF is a major autocrine growth factor induced by TGF-ß. Lu et al. [69] observed that in spontaneous hypertensive rats, CTGF, collagen III, and α-SMA were highly expressed and deposited in glomerular and tubular epithelial cells. These authors suggest that a disrupted interaction between podocytes and CTGF produces this deposition by transforming epithelial and interstitial cells into myofibroblasts, leading to interstitial fibrosis with glomerulosclerosis.

Clinical studies suggested that inhibition of TGF-β/Smad and CTGF pathways could be potential targets in the treatment of glomerular diseases. When patients with FSGS are treated with TGF-β promoters blocking the small synthetic molecule, pirfenidone, GFR loss is decreased by 25% [70]. In contrast, fresolimumab, a high-affinity neutralizing antibody that targets TGF-β isoforms, has no benefit on partial or complete disease remission in patients with FSGS [71]. However, in a clinical study, the inhibition of CTGF, using a human monoclonal antibody, FG-3019, in patients with DKD decreased albuminuria [72].

ELMO1-TGF-β also increases the expression of ELMO1 in diabetic mice, progressively enhancing oxidative stress and increasing the plasma levels of TGF-β. Thus, a feedback mechanism of TGF-β and ELMO1 expression plays a key role in renal tubules [73]. In vitro overexpression of ELMO1 in cultured COS cells increases the expression of TGF-B1 and its downstream ECM genes, including fibronectin, collagen 1A1, and integrin-linked kinase [74]. These increased expressions lead to excessive accumulation of ECM proteins and thickening of the GBM in fibrogenesis. These processes result in the initiation and progression of diabetic glomerulosclerosis [65,75]. Thus, ELMO1 inhibition could be considered a therapeutic strategy.

Adiponectin (ADIPOQ): Adiponectin is produced in adipose tissue as an important peptide hormone involved in glucose regulation and fatty acid catabolism. Decreased adiponectin levels result in oxidative stress, the fusion of podocyte foot processes, and microalbuminuria [76]. In patients with macroalbuminuria, progression to ESRD is found to be associated with higher serum adiponectin levels [77]. Adiponectin knockout mice showed increased susceptibility to podocyte injury in progressive renal diseases [78]. Thus, it could be a potential biomarker for kidney damage and a useful therapeutic target for DKD. Stimulation of the adiponectin receptor in podocytes yielded activation of AMPK that increased cellular uptake of glucose [79]. AdipoRon, an orally active synthetic adiponectin receptor agonist, ameliorated insulin resistance and reversed diabetes in db/db mice with DN specific renal features [80]. Although AdipoRon has an improved safety profile, further studies are needed to confirm its clinical efficacy and additional safety [81].

IRAK4 and TRAF6: When STZ-injected DN model mice were treated with Ellagic Acid (EA), IRAK4 and TRAF6 expressions were inhibited by TLR4, IKK-β, NF-KBp65, and HMGB1. Simultaneously, body weight, blood glucose, serum albumin, serum TNFα, renal function, and antioxidative enzymes of these mice were restored. These results suggest that EA ameliorated STZ-induced oxidative renal injury by inhibition of the HMGB1-TLR4-NF-KB pathway [82]. In another study, EA also significantly reduced the serum levels of proinflammatory cytokines, IL1B and IL6, and reversed STZ-induced DN pathophysiology [83].

Erythropoietin (EPO): Renal anemia is a condition of kidney disease due to the deficiency of EPO. Both alterations in the function of EPO-producing cells and perturbations of the oxygen-sensing mechanism in the kidney may contribute to the development of damaged kidneys [84]. Renal EPO (REP)-producing cells are peritubular interstitial cells that are distributed all over the renal cortex. EPO-producing ability is decreased in REP and contributes to renal fibrosis [85]. Neuroprotective agents, dexamethasone, and neurotrophins in agreement with the neural crest origin of REP cells restore EPO production and alleviate renal fibrosis.

Bcl2-Bcl2 plays an important role in podocyte damage and the accumulation of ECM, leading to further glomerulosclerosis [86]. In DN model mice, cell apoptosis was significantly increased, and Bcl2 was significantly decreased. Anthocyanins (Grape Seed Procyanidin (GSPE)) activate the expression of Bcl2 in diabetic mouse kidneys to suppress renal cell apoptosis. This study demonstrates that anthocyanins may exhibit protective effects against HG-induced renal injury in DN [87].

Vitamin D Receptor (VDR): Vitamin D exerts renoprotective actions by transcriptionally suppressing renin [88]. Combination therapy with an Angiotensin Receptor Type 1AT1 (AT1) receptor blocker and a vitamin D analog markedly ameliorated renal injury in STZ-induced diabetes mice by blocking the renin rise. These combined treatments suppressed the induction of fibronectin, TGF-β, and MCP-1 and reversed the decline of slit diaphragm proteins nephrin, Neph-1, ZO-1, and α-actinin-4. Vitamin D alone can directly suppress the HG induction of TGF-β and MCP-1 in mesangial cells by blocking Ang II accumulation [89].

### Inflammatory Genes for Fibrotic Changes in DN

Fibrosis is the excessive production and deposition of ECM protein in the kidney tissues and is essentially activated by inflammation. Thus, progressive fibrosis with sustained inflammation can be considered a chronic defect in wound healing and tissue repair [90].

MIF1: Macrophage Inhibitory Factor 1 (MIF1) is a proinflammatory cytokine. A marked increase in both glomerular, tubular MIF1 mRNA and protein expression were observed in proliferative forms of Glomerulonephritis (GN) correlating with leukocyte infiltration, histologic damage, and renal function impairment [91]. Treatment of diabetic db/db mice with the MIF1 inhibitor, ISO-1, significantly decreased blood glucose levels and albuminuria and promoted wound healing in kidney tissues, suggesting that MIF1 inhibition may be a potential therapeutic strategy in DN.

MCP1(CCL2, MCP-1; Monocyte Chemoattractant Protein-1 (MCP-1): In MCP-1 (−/−) db/db mice, kidney macrophage accumulation and the progression of diabetic renal injury were substantially reduced compared to MCP-1(+/+) db/db mice with equivalent diabetic conditions [92]. Strong upregulation of MCP-1 was observed in tubular cells in biopsies from patients with T2D with nephropathy, correlating NFKB1 activation [93]. Urinary MCP-1 levels were found significantly elevated in patients with DN and well correlated with the number of CD68-positive infiltrating cells in the interstitium [94]. Inhibition of MCP-1 expression with various agents, such as methanolic extract from unripe kiwi fruit (*Actinidia deliciosa*), dehydroabietic acid, capsaicin, curcumin modulates NFKB1, TNFα, nitric oxide, IL8, and adiponectin resulted in a reduction of DN pathogenesis with a subsequent reduction in tissue damage [95].

IL1A, IL1B, IL1RN, IL6, IL8, IL10, IL18, IL100: Infiltration of macrophages and lymphocytes and the overproduction of proinflammatory chemokines and cytokines are observed in renal diabetic tissues [96]. Fibroblasts can be derived from endothelial EMT and EMT development is associated with IL1, IL6, IL18, and TNFα [90]. Pentosan Polysulfate (PPS) largely attenuates the inflammation of nephropathy in aging diabetic mice. When HG-containing HK-2 cells were incubated with PPS, inhibition of the p38 MAPK pathway and reduction of proinflammatory cytokines, such as TNFα, IL1B, and IL6, were observed. PPS markedly suppressed the HG-induced reduction in cell viability and ameliorated p38 MAPK-mediated renal cell apoptosis and inflammation [97].

In mice with diabetes-induced nephropathy, Ursolic Acid (UA) reduced kidney/body weight index, protected kidney cells, and alleviated inflammation by reducing TNFα, IL1β, IL 6, and IL 18 levels and kidney cell damage. UA also suppressed TLR4, MyD88, and NFKB1 protein expression, suggesting that it had the potential to be a therapeutic agent against DN [98]. Shukla et al., 2018 [99] showed that pty-2 (extract from *Pueraria tuberosa*) decreased the expression of IL6 and TNFα (both were elevated in DN) and modulated the expression of NFKB1, suggesting that it could be used as a therapeutic agent for reversing DN.

Mononuclear macrophages play a significant role in the development of DN. These are the major immune cells responsible for renal tissue stromal hyperplasia, glomerular sclerosis, and irreversible pathological changes of glomeruli [100,101]. Specifically, they are of two types in terms of their functions: classical M1 macrophages activated by Th1 cytokines or lipopolysaccharides and M2 macrophages activated by Th2 cytokines. Under the HG condition, M1 macrophage decreases the expression of Sirt6 that inhibits M1/M2 transformation, leading to podocyte apoptosis in a dose-dependent manner [102].

Lu et al., 2017 [103] suggest that curcumin is a promising treatment for DN. Its renoprotective effects occur by the inhibition of IL1B expression, NLRP3 inflammasome activity, and cleavage of caspase 1. Correa-Costa et al., 2011 [104] confirmed the important roles of both TLRs and NLRP3 inflammasomes by allopurinol, which downregulated components of the inflammasome pathway and diminished cell injury. Shahzad et al. [105] reported NLRP3 activation in podocytes in a murine DKD model. They demonstrated that increased IL1B and IL18 expression in the plasma and renal cortex of diabetic animals are correlated with urine albumin/creatinine ratio, which is a functional kidney biomarker. Glibencamide improved renal function and ameliorated CKD histopathology in an adenine-rich diet rat model of CKD that attenuated NLRP3 expression [106]. One of the sulfonylurea-containing compounds, derived from glyburide, MCC950 (also CP-456,773), was reported as a potent specific inhibitor of the NLRP3 inflammasome [107]. These results strongly indicate that the NLRP3 inflammasome is a potential therapeutic target in DN.

IL1RA is increased with disease progression until stage 4–5, with a loss of functional renal mass that occurs in late-stage CKD. Overexpression of IL1RN increased the mean serum level of IL1RA after temporary xenotransplantation, with a significant reduction of hyperglycemia. A mildly elevated serum concentration of IL1RA protected and enhanced engraftment of islet isografts immediately after transplantation [108].

The IL8 T251A variant lies in the regulatory region of the gene and increases its expression. Increased urinary excretion of IL8 has been reported in DN patients [109]. The levels of urinary IL8 in patients with DN increased in the second and third stages of pathogenesis. There was a significant correlation between the levels of urinary IL8 and HbA1c. HG may stimulate IL8 production and their excretion into the urine independently, which creates pathological lesions of the disease [109].

TNFα and TNFRSF19: The pathogenic role of TNFα is observed in models of immune-complex-mediated glomerulonephritis, lupus nephritis, Antineutrophil Cytoplasmic Antibody (ANCA)-associated glomerulonephritis, DN, Acute Kidney Injury (AKI), and kidney allograft rejection [110]. TNFα-/- knockout mice showed a reduction in the severity of antiglomerular basement antibody-induced nephritis [111]. These chimeric mice also showed bone-marrow-derived intrinsic renal cells without TNFα. These studies suggest that intrinsic renal cells are the major contributor to TNFα-mediated tissue injury. Khan et al., 2005 [112] demonstrated that neutralization of endogenous TNFα reduced glomerular inflammation, crescent formation, and tubulointerstitial scarring with the preservation of renal function. TNFα blockade was effective even when introduced at the time of maximum glomerular inflammation.

The TNF Receptor Superfamily (TNFRSF) includes receptors for TNFSF ligands. Most TNFSF ligands modulate cell proliferation, survival, differentiation, and apoptosis in kidney cells [113], although the exact role of TNFSFR19 in DN is not precisely established.

IFN Gamma (IFNG): Elevated levels of proinflammatory cytokine, IFNG, are observed in nephropathic patients and involved in nephropathy complication of T2D [114]. Wang et al., 2012 [115] observed that when splenocytes cultured with Ad-IκBα-IRES2-CD40L-transfected islet grafts were transferred to diabetic rats, IFNG was reduced in peripheral blood. Bai et al., 2017 [116] demonstrated that Mesenchymal Stem Cell (MSC) treatment proved to be effective in DN models by protecting renal function and preventing fibrosis through Lipoxin A4 (LXA4).

CCR5: CCR5 59029G/A polymorphism is significantly related to enhanced susceptibility to DN with T2D [117]. Fibrocytes also have the ability to produce certain chemokine receptors, such as CCr2, CCr3, CCR5, and CXCR4. As a GPCR-β chemokine factor, CCR5 plays a vital role in the chemotaxis, proliferation, and immunoregulation of inflammatory cells [118]. Combined inhibition of CCR2 and CCR5 receptors may decrease albuminuria and prevent kidney function decline in patients with DN. CCR2 blockade with an orally available small-molecule antagonist, RO5234444, alleviates proteinuria, glomerulosclerosis, and kidney failure in diabetic db/db mice [119]. Gale et al., 2018 [120] indicated a modest effect of PF-04634817 in reducing albuminuria in T2D patients who received SOC treatment.

MMP9: Renal interstitial fibroblasts induce the expression of MMP9. DN is characterized by excessive deposition of ECM protein and disruption of the glomerular filtration barrier. In STZ-induced diabetes, MMP9-overexpressed mice developed markedly increased albuminuria, glomerular hypertrophy, and thickening of the GBM [121]. Overexpression of endogenous MMP9 induced podocyte dedifferentiation and promoted podocyte monolayer permeability to albumin with ECM synthesis. Li et al., 2008 [122] showed that TGF-β1 was able to stimulate podocyte MMP9 secretion. These findings indicate that an increase in intraglomerular MMP9 is common during albuminuria in DN and explain the reason why TGF-β-neutralizing antibody attenuates glomerular sclerosis but not albuminuria [121]. Kundu et al., 2013 [123] showed that MMP9 played a critical role in regulating H_2_S production in the diabetic condition and diabetic renal remodeling. Masola et al., 2012 [124] showed that sulodexide was an effective heparanase-1 inhibitor that increased MMP9-mediated switching of the autocrine loop of FGF-2. As FGF2 activates the progression of nephropathy to renal failure, it supports the conviction that sulodexide can protect against renal fibrosis sustained by EMT, thereby preventing the progression of DN to ESRD.

MMP3 and MMP12: Tubulointerstitial injury in progressive renal impairment involves human Proximal Tubular Epithelial Cells (PTECs). Synthetic MMP3 inhibitors or MMP3 gene knockdown by siRNA revealed that the constitutive and accelerated shedding of KIM-1 in cultured PTEC [125]. During the progression of nephropathy, MMP3 levels in serum increases in patients with macroalbuminuria [126]. MMP3 has a direct correlation with GFR, which is independent of protein excretion in macroalbuminuria. Thus, MMP3 activity has a correlation with disease progression toward end stages, and its inhibitors could be developed for therapy.

## 8. Conclusions

DN is a devastating disease with progressive consequences, and about half of diabetes patients exhibit ESRD in their lifetime. Therefore, preventing ESRD is of prime importance to control diabetes-related renal complications. Although much research for early-stage diagnosis and drug development efforts are devoted to this area, current preventive and curative measures for renal tubule ailments are far from satisfactory. This is due to the complex nature of the disease, as many factors affect the development of ESRD in DN. Regardless, it is considered an inflammatory progression of diseases, and consequently, mutations in numerous inflammatory genes are identified and associated with DN. Inflammatory and cytokine molecules regulate prominent genes whose products are needed to develop lesions and facilitate the progression of kidney abnormalities. For developing genomic medicine, inflammatory genes may be targeted by manipulating their mutations or even by reverting to wild type with genome corrections.

## Data Availability

All data will be available upon publication.

## Abbreviations

AMP—Adenosine Monophosphate; AT1—Angiotensin Type 1; DN—Diabetic Nephropathy; CKD—Chronic Kidney Disease; CMA—Chaperone-Mediated Autophagy; DCCT—Diabetes Control and Complications Trial; DKD—Diabetic Kidney Disease; ECM—Extracellular Matrix; EA—Ellagic Acid; EDNRA—Endothelin-1 (EDN1)/Endothelin Receptor Type A; EMT—Epithelial-to-Mesenchymal Transition; ESRD—End-Stage Renal disorders; EDNRA—Endothelin Receptor Type A; FSGS—Focal Segmental Glomerulosclerosis; GAP—GTPase-Activating Protein; GBM—Glomerular Basement Membrane; GFR—Glomerular Filtration Rate; GN—Glomerulonephritis; HG—High Glucose, IFTA—Interstitial Fibrosis and Tubular Atrophy; NAP—Nonalbumin Proteinuria; RAS—Renin–Angiotensin System; STZ—Streptozotocin; SOC—Standard Of Care; UA—Ursolic Acid; T1D—Type 1 Diabetes; T2D—Type 2 diabetes.
